# Progressive reorganization of mitochondrial apparatus in aging skeletal muscle of naked mole rats (*Heterocephalus glaber)* as revealed by electron microscopy: potential role in continual maintenance of muscle activity

**DOI:** 10.18632/aging.203720

**Published:** 2021-11-28

**Authors:** Valeriya Vays, Irina Vangely, Chupalav Eldarov, Susanne Holtze, Thomas Hildebrandt, Lora Bakeeva, Vladimir Skulachev

**Affiliations:** 1 Lomonosov Moscow State University, Belozersky Research Institute of Physico-Chemical Biology, Moscow 119991, Russia; 2 Department of Reproduction Management, Leibniz-Institute for Zoo and Wildlife Research, Berlin 10315, Germany

**Keywords:** aging, neoteny, mitochondria, naked mole-rat, electron microscopy

## Abstract

The authors examined the ultrastructure of mitochondrial apparatus of skeletal muscles of naked mole rats (*Heterocephalus glaber*) from the age of 6 months to 11 years. The obtained results have demonstrated that the mitochondria in skeletal muscles of naked mole rats aged below 5 years is not well-developed and represented by few separate small mitochondria. Mitochondrial reticulum is absent. Starting from the age of 5 years, a powerful mitochondrial structure is developed. By the age of 11 years, it become obvious that the mitochondrial apparatus formed differs from that in the skeletal muscle of adult rats and mice, but resembles that of cardiomyocytes of rats or naked mole rats cardiomyocytes. From the age of 6 months to 11 years, percentage area of mitochondria in the skeletal muscle of naked mole rat is increasing by five times. The growth of mitochondria is mainly driven by increased number of organelles. Such significant growth of mitochondria is not associated with any abnormal changes in mitochondrial ultrastructure.

We suppose that specific structure of mitochondrial apparatus developed in the skeletal muscle of naked mole rats by the age of 11 years is necessary for continual skeletal muscle activity of these small mammals burrowing very long holes in stony earth, resembling continual activity of heart muscle. In any case, ontogenesis of naked mole rat skeletal muscles is much slower than of rats and mice (one more example of neoteny).

## INTRODUCTION

Aging is considered as a complicated physiological process associated with significantly decreased neuromuscular function and muscular performance capability, accompanied by structural disorganization of muscle tissue [1–8]. Mitochondria play a key role in the process of physiological aging and development of age-dependent abnormalities [9–17]. It is mitochondria that undergo the greatest structural changes in the process of aging [18]. Age-dependent changes of mitochondrial ultrastructure have been studied for many years [19–27]. Only one model (flight muscle of insects) is widely known in which unique and specific age-dependent changes in mitochondrial ultrastructure were detected and their functional significance demonstrated. In the classic study of Sacktor and Shimada [28] of age-dependent changes in mitochondrial morphology of the flight muscle of *Phormia regina* blowfly, the authors described local reorganization of inner mitochondrial membrane into myelin-like structures progressively occupying the entire space of mitochondria leading to their structural damage. Based on the contemporary view of mechanisms of aging, Walker and Benzer [29] experimentally demonstrated the key influence of oxidative stress on the development of these age-dependent changes in the ultrastructure of flight muscle mitochondria in Drosophila.

At present, investigation of processes of aging focuses on species with naturally delayed aging. One of the representatives of such species is naked mole rat (*Heterocephalus glaber*). This is a miniature (up to 35 g) rodent living in underground tunnels in arid and semiarid zones of Kenia, Somalia and Ethiopia [30]. One of the most interesting features of naked mole rat is its very long lifespan. The longevity record for the captured naked mole rat is currently over 31 years [31]. The available literature lacks data on the ultrastructural study of naked mole rat tissues except for the study by Onyango et al. [32] performed using the testis of naked mole rat, and the work of Stoll et al. [33] in which selected electron microscopic images are provided for illustration of histological and functional characteristics of naked mole rat. Our investigations [34] of the mitochondrial ultrastructure of cardiomyocytes of naked mole rat demonstrated that by the age of 11 years their mitochondria do not show any abnormalities. The mitochondrial ultrastructure corresponds to the phenotype of young animal which is one of the neotenic features in naked mole rat.

**Figure 1:**
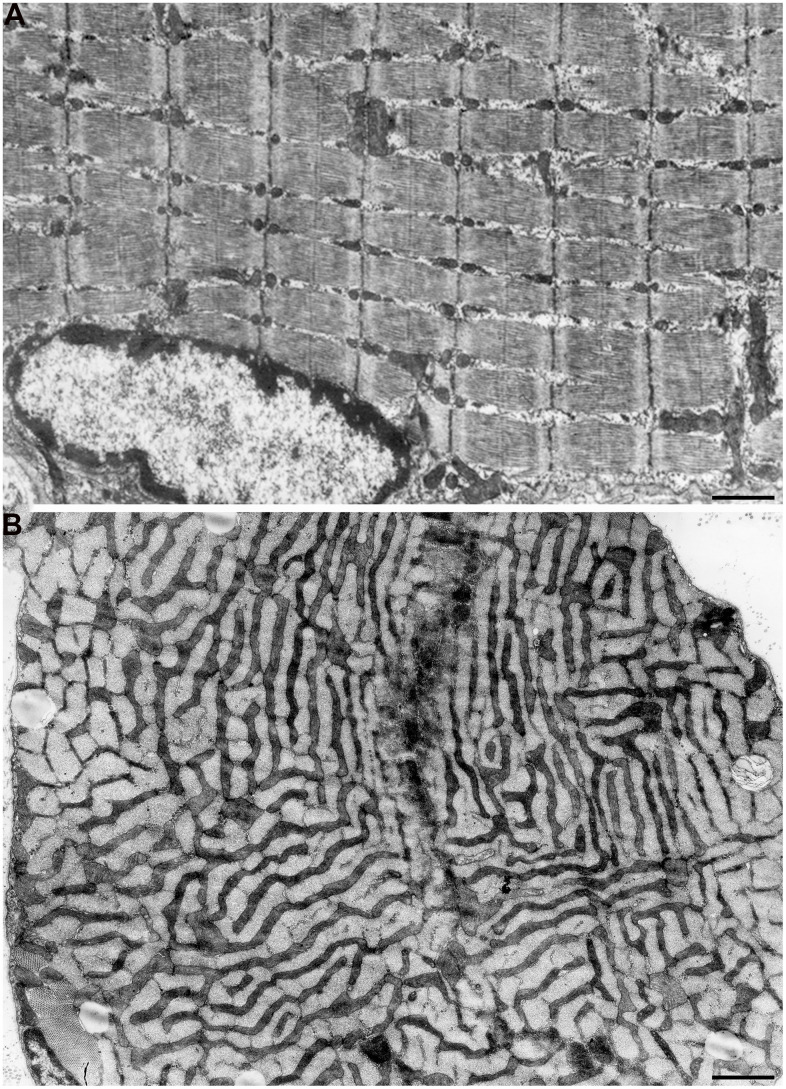
**Mitochondrial reticulum in diaphragm of a two-month-old rat.** (**A**) Longitudinal section. (**B**) Cross section through isotropic region. (from Bakeeva et al. [40]).

According to the literature, mitochondrial apparatus of skeletal muscle fibers has complicated structural organization. As was shown in one of the first electron microscopic studios performed by Palade [35], mitochondria were shown to be arranged in rings or braces around the I bands of myofibrils and have stellate form. Later, Gauthier and Padikula [36] and independently Bubenzer [37], based on the analysis of separate sections of the rat diaphragm, suggested that there are three types of mitochondria in the skeletal muscles: 1) thin, branched, located across the muscle fibers; 2) thicker, oval-shaped, located along the muscle fibers; 3) spherical, located close to the cell ridges with offshoots leading to the cell center. Using multiple three-dimensional reconstructions, we demonstrated that all mitochondrial material in the diaphragm muscles is arranged through specific intermitochondrial contacts in a single mitochondrial network. Such a network is formed by giant, branched mitochondria present on both sides of the Z line and joined into single mitochondrial carcass by longitudinal mitochondria strands located along myofibril bundles. We defined this system as *mitochondrial reticulum* (Figure 1A, 1B) [38–40]. Using high-voltage electron microscopy, Kirkwood et al. [41] examined native tissue of three rat skeletal muscle fiber types and showed that mitochondrial reticulum is a structure existing *in vivo* and not a result of the fixation process or muscle tissue stagnation. Modern scanning microscopy have recently allowed to get a vivid, three-dimensional image of mitochondrial reticulum ultrastructure in mouse skeletal muscles (Supplementary Figure 1) [42]. At the same time, some authors considered the branched network detected in isolated sections of skeletal muscles and formed by elongated mitochondria as a result of congenital myopathy [43] or as a sign of aging [44].

We have studied skeletal muscle tissue of naked mole rat in order to detect specific age-dependent changes in the ultrastructure of mitochondrial apparatus. This work is a follow-up study to our previous research [17, 45]. The present paper describes the results of the study of the mitochondrial ultrastructure of the naked mole rat skeletal muscles of the following age groups: 1 week, 6 months, 5 years, 7 years and 11 years.

## RESULTS AND DISCUSSION

**Figure 2:**
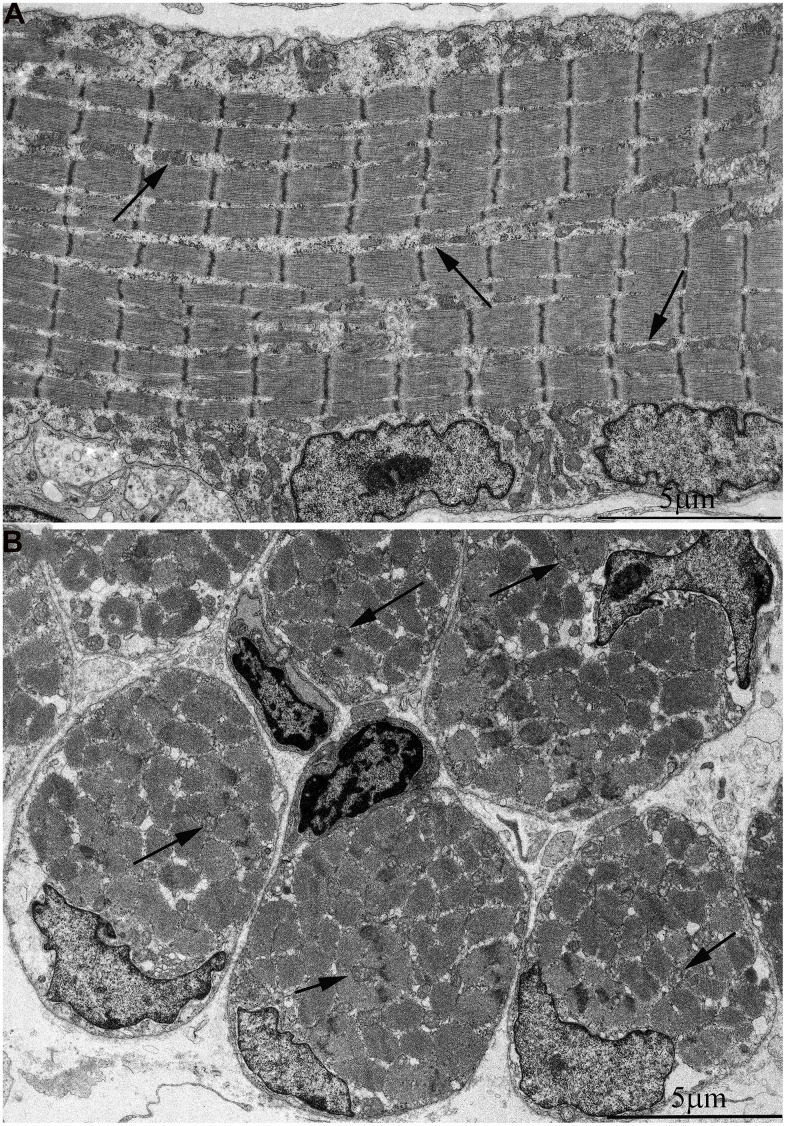
**Ultrastructure of mitochondria in skeletal muscle of one-week-old naked mole rat.** (**A**) Longitudinal section. Arrows indicate mitochondria. (**B**) Cross section. Widely spaced, small mitochondria are observed on the longitudinal and cross section of muscle fiber. Arrows indicate mitochondria.

**Figure 3:**
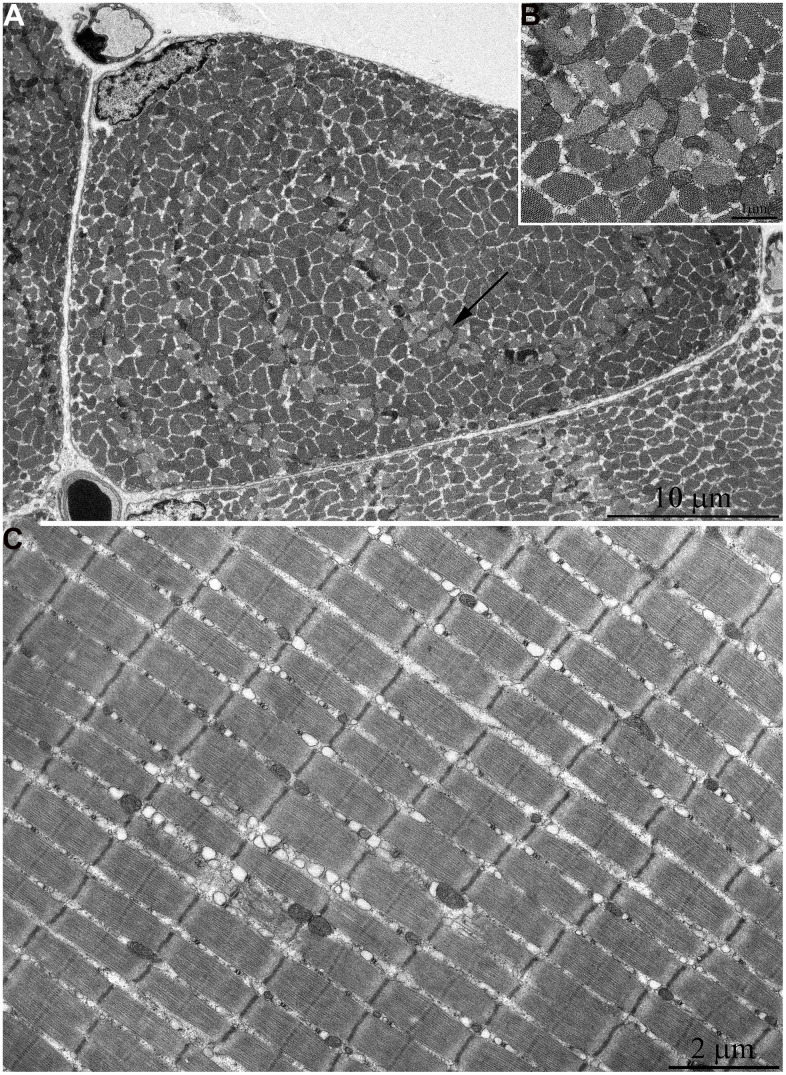
**Ultrastructure of mitochondria in skeletal muscle of six-month-old naked mole rat.** (**A**) Cross section of muscle fiber. Small, isolated mitochondria and group of mitochondria, which ultrastructure is demonstrated at higher magnification in (**B**) is indicated by an arrow. (**C**) Longitudinal section of muscle fiber. Small, widely spaced mitochondria.

**Figure 4:**
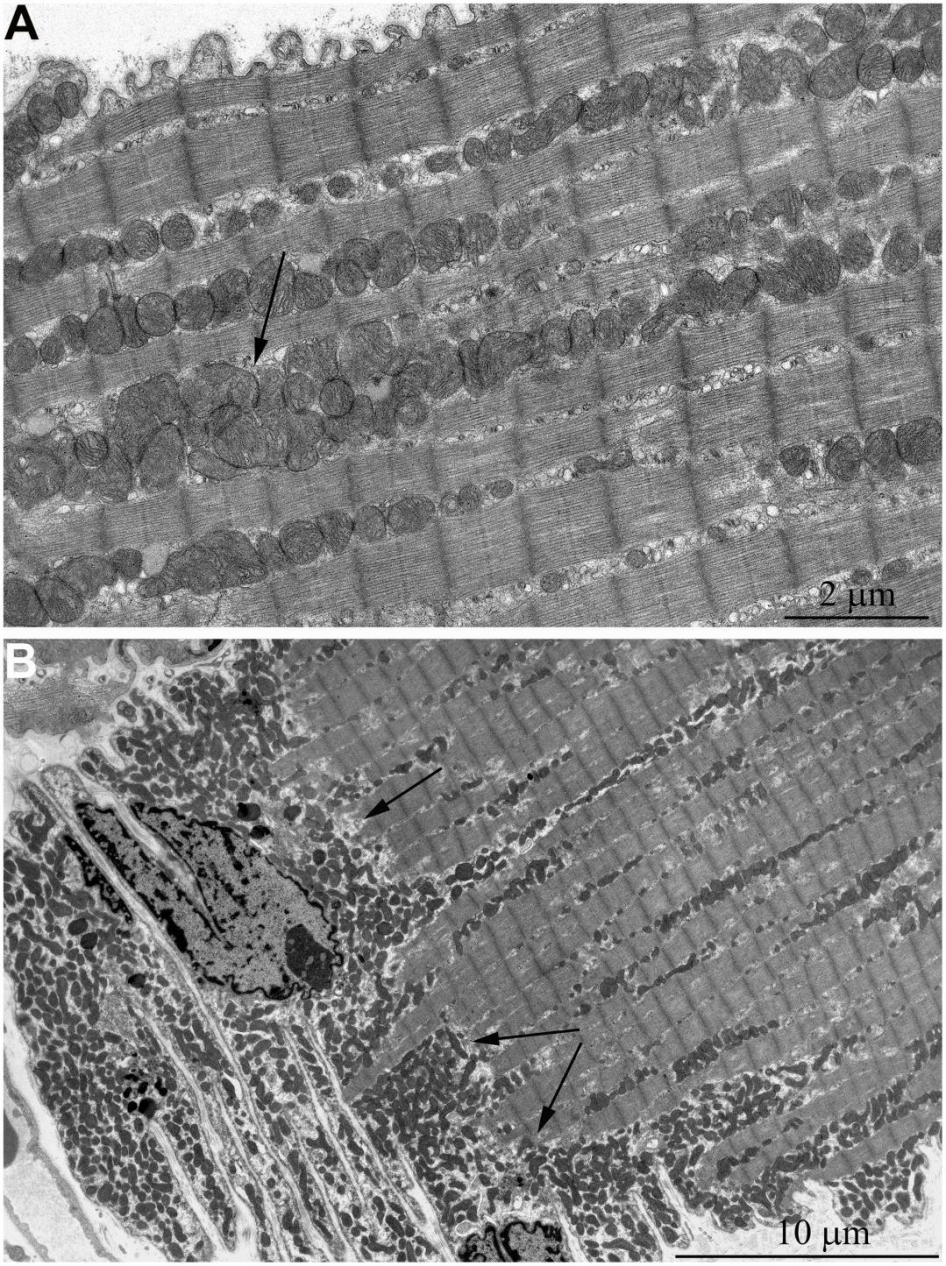
**Ultrastructure of mitochondria in skeletal muscle of 5-year-old naked mole rat.** (**A**) Longitudinal section of muscle fiber. Rows of mitochondria arranged along myofibrils can be observed, mitochondrial cluster is indicated by arrow. (**B**) Longitudinal section of muscle fiber. Large clusters of mitochondria in the perinuclear and subsarcolemmal areas are indicated by arrows.

In Figure 2A, 2B, the ultrastructure of mitochondria in skeletal muscle of naked mole rat at the age 1 week is presented. At the longitudinal (Figure 2A) and cross sections (Figure 2B) of the muscle fiber small, widely spaced mitochondria can be seen. In our previous studies [40], the number of mitochondria in the skeletal muscles of rats has been reported to increase significantly shortly after birth. Moreover, mitochondria demonstrated stepwise fusion and formation of three-dimensional reticulum. This process is completed by the age of 1.5-2 months. As for mitochondria of 6-month-old naked mole rats they were still small and isolated from neighbors in both cross and longitudinal sections of the muscle fiber (Figure 3A–3C). By the age of 5 years, the ultrastructure of mitochondrial apparatus of naked mole rat undergoes acute changes. It can be clearly seen in Figure 4 that the number and size of mitochondria became significantly higher, mitochondrial clusters have appeared not only in the perinuclear and subsarcolemmal areas but also between myofibrils (indicated by arrows in Figure 4A, 4B). However, mitochondrial network is not formed.

**Figure 5:**
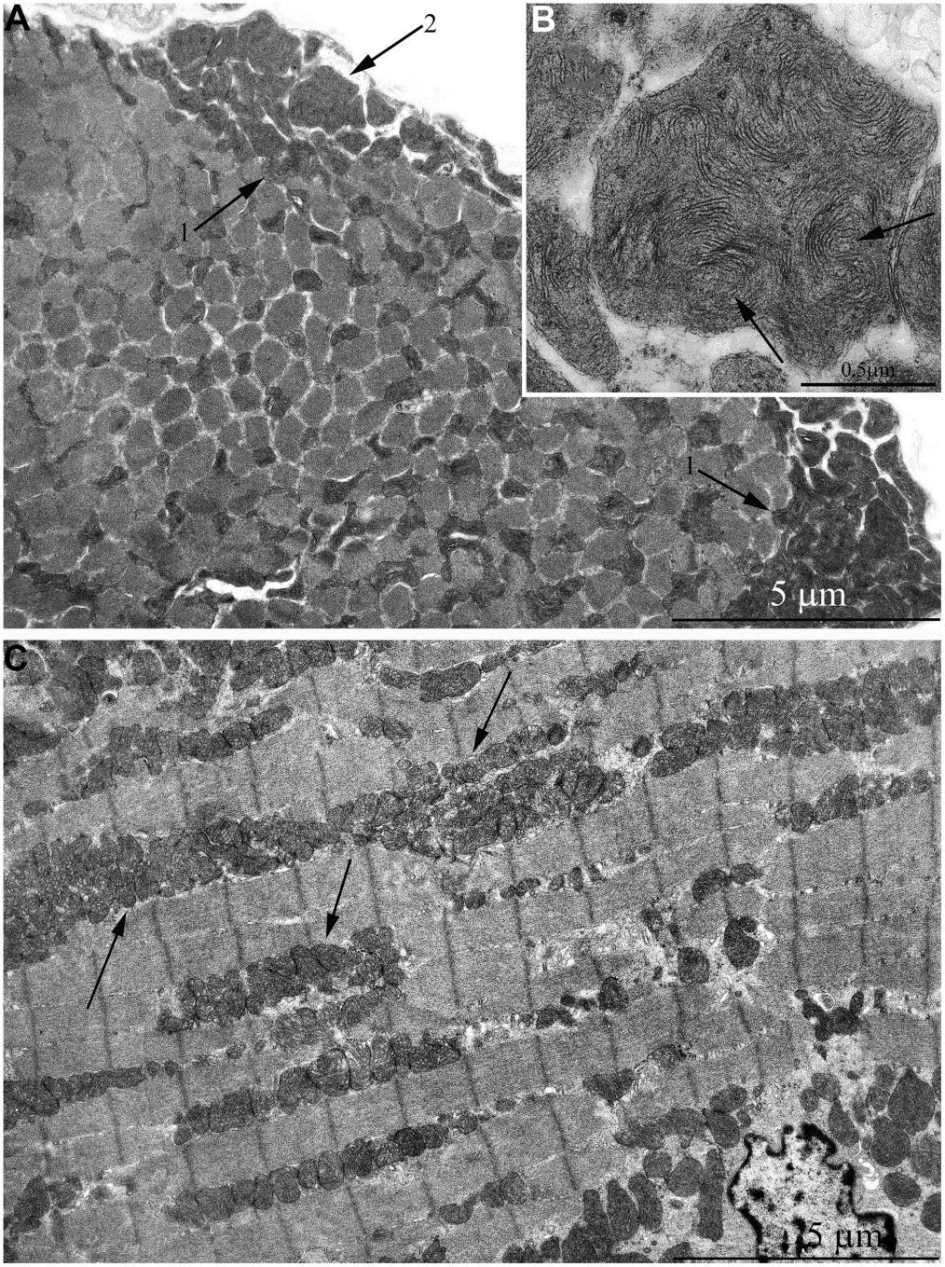
**Ultrastructure of mitochondria in skeletal muscle of 7-year-old naked mole rat.** (**A**) Cross section of muscle fiber. Clusters of large mitochondria in the subsarcolemmal area are indicated by arrows 1. Mitochondrion of specific ultrastructure, which is demonstrated in Figure 5B, is indicated by arrow 2. (**B**) Mitochondrion which specific ultrastructure, cristae in form of curled, wave-like structures are indicated by arrows. (**C**) Longitudinal section of muscle fiber, large clusters of mitochondria localized along myofibrils are indicated by arrows.

At the age of 7 years, further increase of number and size of mitochondrial clusters located along myofibrils (indicated by arrows in Figure 5C) and in the subsarcolemmal area (arrow 1 in Figure 5A) were observed. Besides, very large isolated mitochondria appear in the subsarcolemmal area with non-typical morphology for skeletal muscle (Figure 5A, arrow 2). Figure 5B shows an image of the same mitochondrion at higher magnification. The whole intermembrane space of the mitochondrion is filled with cristae in the form of curled, wave-like structures (indicated by arrows). It should be noted that previously we found mitochondria of similar ultrastructure in cardiomyocytes of naked mole rat aged above 5 years [34].

**Figure 6:**
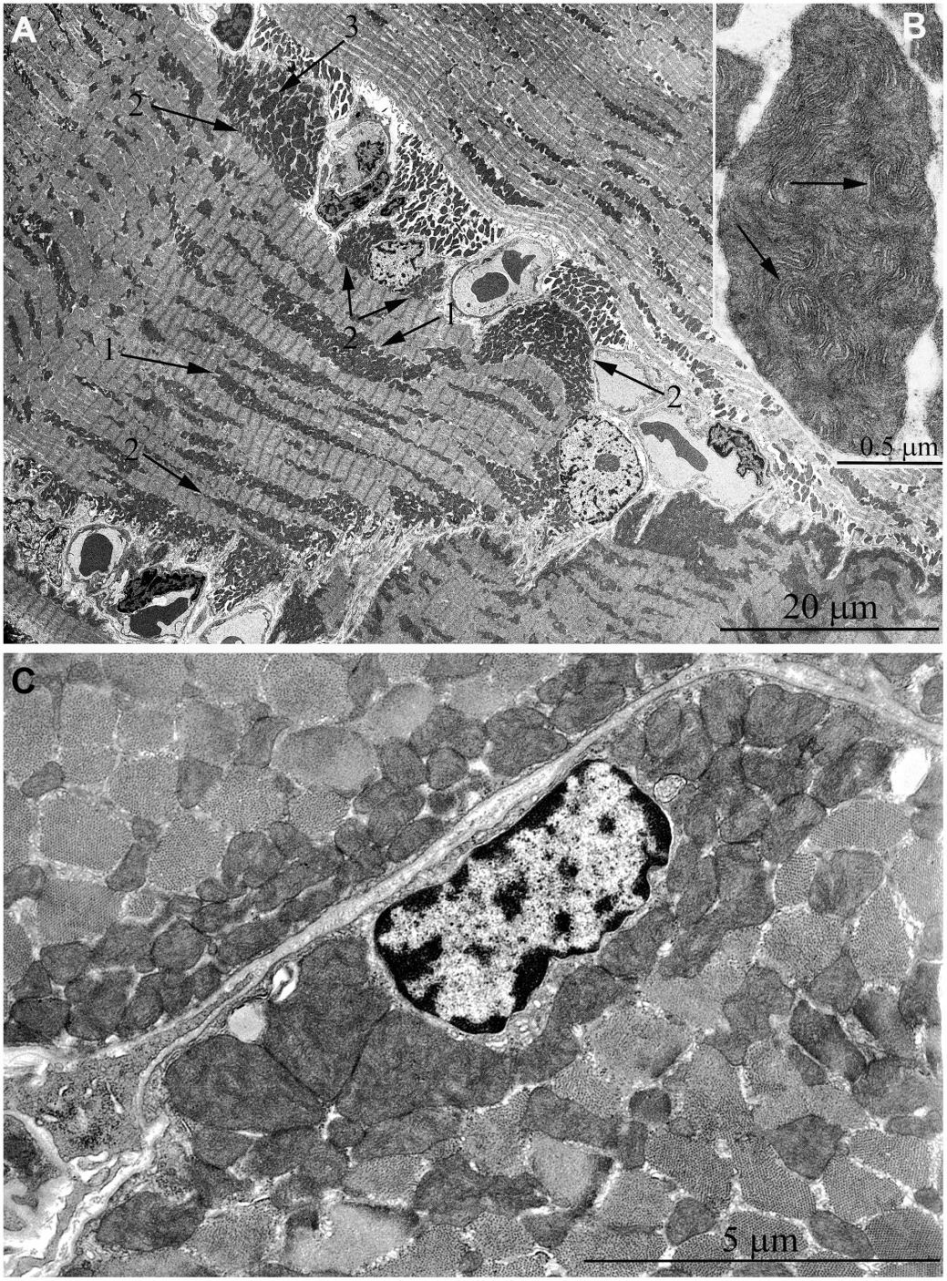
**Ultrastructure of mitochondria in skeletal muscle of 11-year-old naked mole rat.** (**A**) Longitudinal section of muscle fiber. Arrows 1 indicate large clusters of mitochondria located along myofibrils; arrows 2 indicate large clusters of mitochondria in the perinuclear and subsarcolemmal areas. Mitochondrion which specific ultrastructure is demonstrated under higher magnification in Figure 6B is indicated by arrow 3. (**B**) Mitochondrion which specific ultrastructure, convoluted stacks of cristae are indicated by arrows. (**C**) Cross section of muscle fiber. Similar ultrastructural pattern in the cross-section of muscle fiber is typical of cross-sections of cardiomyocytes, excluding subsarcolemmal localization of the nucleus.

The same trend in the development of mitochondrial apparatus in skeletal muscle was detected in animals aged 11 years. The characteristic feature of the ultrastructure of skeletal muscle fibers in this group of animals is very large mitochondrial clusters both in the subsarcolemmal area and between myofibrils (indicated by arrows 1 and 2 in Figure 6A).

**Figure 7:**
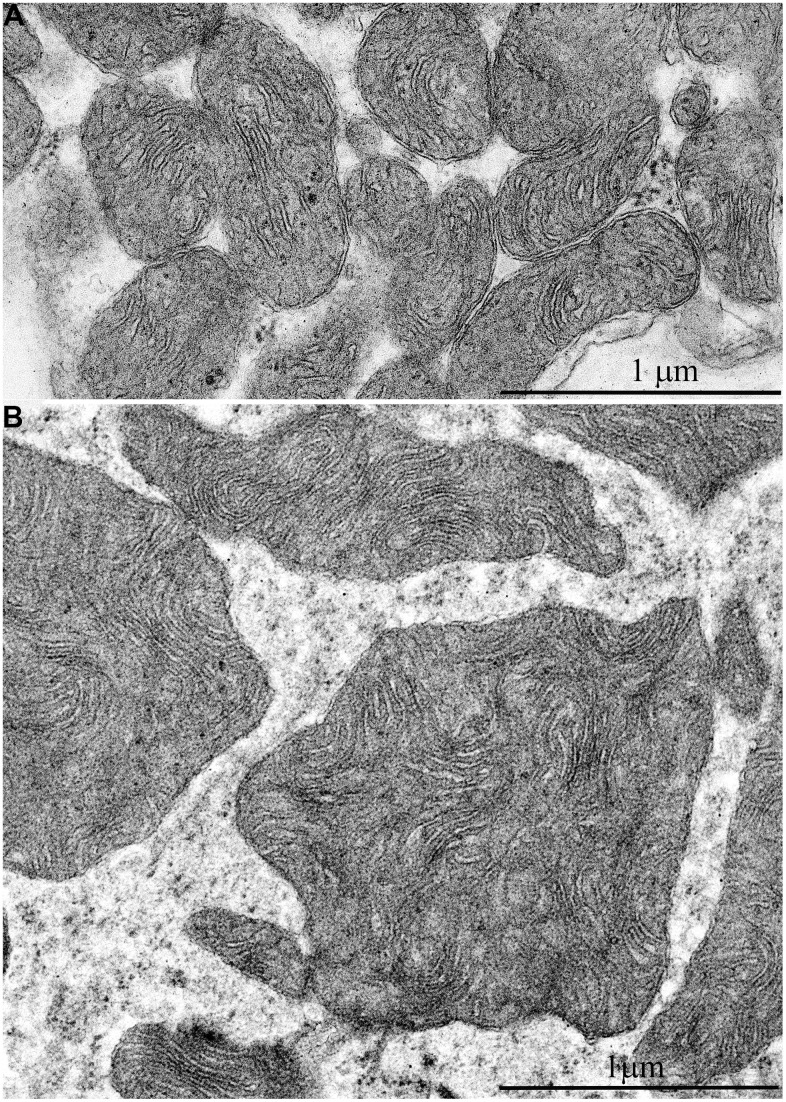
Comparison of ultrastructures of mitochondria in skeletal muscle fibers of naked mole rats of two different ages is shown at the same magnification: (**A**) Skeletal muscle mitochondria at the age of 6 months; (**B**) Skeletal muscle mitochondria at the age of 11 years.

In addition, one prominent feature of mitochondrial ultrastructure in this group of naked mole rats is its internal organization which previously has not been observed in skeletal muscles of any animals. Figure 6B shows densely packed, wave-like mitochondrial cristae. Depending on the section plane, it can be seen that cristae are arranged in separate convoluted stacks. In Figure 7A, 7B shows the ratio of size and ultrastructure of mitochondria in skeletal muscle fibers of naked mole rat aged 6 months and 11 years at the same magnification. Significantly increased size of mitochondria, number of cristae, density of the matrix can be observed.

**Figure 8:**
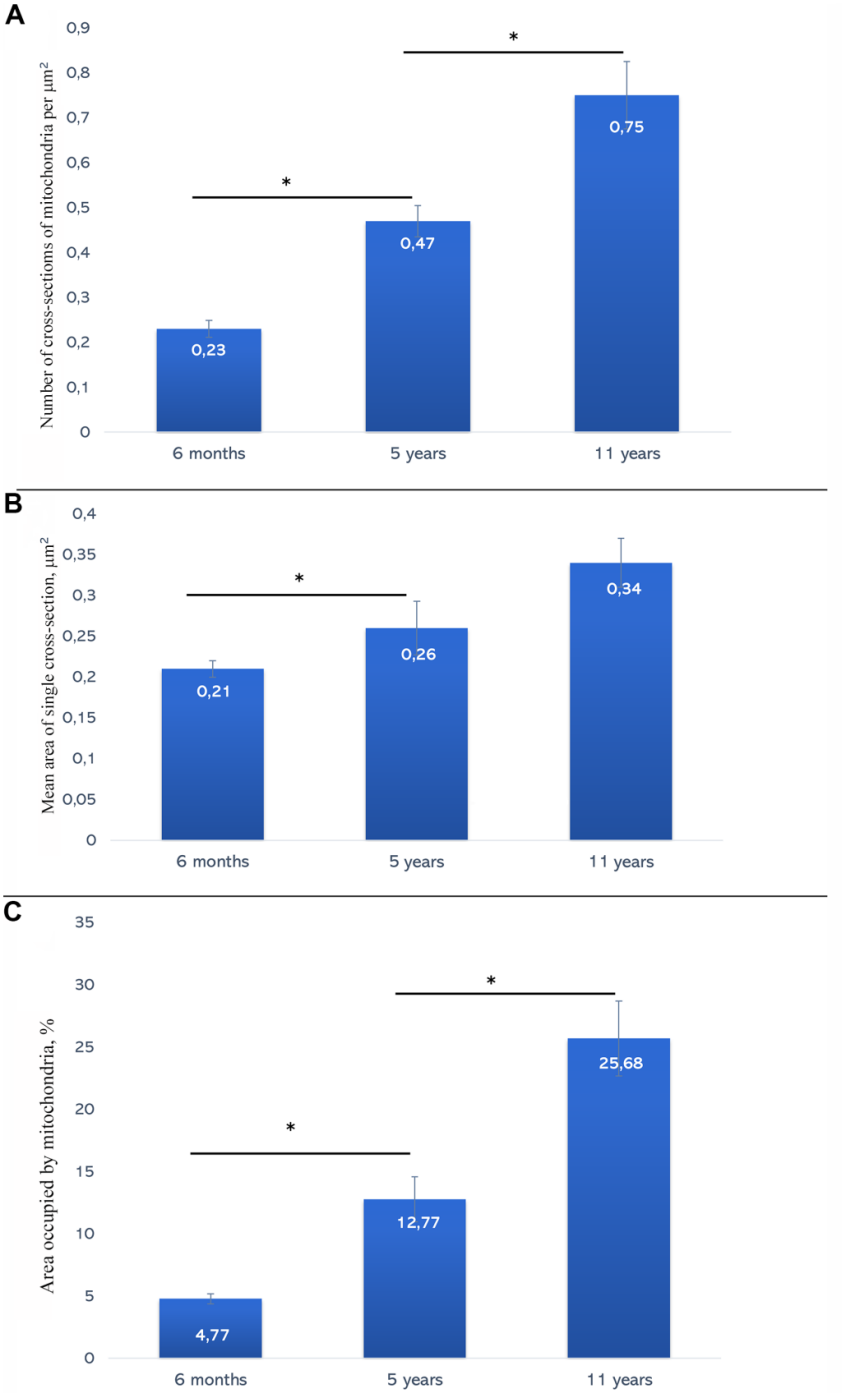
(**A**) Average values of the number of mitochondria per 1 μm^2^ of muscle fiber in naked mole rats of different ages. (**B**) Average values of sectional area of muscle fiber mitochondria in 6-month-old, 5-year-old and 11-year-old naked mole rats. (**C**) Area occupied with mitochondria in muscle fibers in naked mole rats of different ages (%). * The difference is significant at *p* < 0.05. Error bars on all the graphs correspond to the standard error.

Morphometric analysis was performed in order to assess muscle-specific mitochondria development. Figure 8A shows the results of counting the number of mitochondrial sections per 1 μm^2^ of the muscle fiber. At the age of 6 month, mean number of mitochondria in the skeletal muscle were 0.23 ± 0.02 items/μm^2^ with the more than two-fold increase in 5-year-old animals (up to 0.47 ± 0.03 items/μm^2^). The reported differences are significant at p<0.05. In 11-year-old naked mole rat, the number of sections was ever larger (0.75 ± 0.07 items/μm^2^). The differences are significant at p<0.05.

The second parameter characterizing age-dependent changes of the mitochondrial structure of muscle tissue of naked mole rat is the mean area of a single mitochondrion cross-section. In 6-month-old naked mole rats, the value of this parameter was at 0.21 ± 0.01 μm^2^. By the age of 5 years, its value has increased up to 0.26 ± 0.03 μm^2^. The differences are significant at p < 0.05. By the age of 11 years, it increased further, up to 0.34 ± 0.03 μm^2^ (Figure 8B).

Third morphometric parameter calculated was volume fraction of mitochondria from the total volume of muscle fiber (Figure 8C). At the age of 6 months, the fraction was 4.77 ± 0.42%. By the age of 5 years, it has significantly increased by almost three times – up to 12.77 ± 1.81%, p < 0.05. By the age of 11 years, mitochondrial volume fraction of naked mole-rat was found to reach mean value of 25.68 ± 3%, with almost two-fold increase compared to 5-year-old animals.

It should be stressed that by the age of 11 years the general architecture of mitochondria in skeletal muscle of naked mole rat has been found to undergo reorganization with the formation of ultrastructure features typical for cardiomyocytes. Indeed, longitudinal sections of muscle fibers show arrangement of mitochondrial clusters along myofibrils, and cross sections of muscle fibers show a great number of large mitochondria instead of thin elongated organelles (Figure 6C). According to the literary date, similar ultrastructural pattern of the cross section of muscle fiber is typical for cardiomyocytes [46–50]. A question arises why mitochondria in skeletal muscles of adult naked mole rat resemble cardiomyocytes (a bunch of large round mitochondria connected to each other with intermitochondrial four-membrane contact) than mitochondria in rat myocytes (mitochondrial reticulum, i.e. a network of elongate branched organelles). Perhaps, the reason for such a difference is due to continual activity of muscular work in naked mole rats, small animals burrowing very long holes in stony earth. Such a work resembles continual activity of the heart muscle, rather than usual skeletal muscle of, say, Wistar rats, where that activity is alternates with rather long period of the rest.

This finding is especially important as it is completely different to the data on age-dependent ultrastructural changes of the mitochondrial apparatus in skeletal muscles of short-lived rodents. Currently, it is generally accepted that the severity of sarcopenia and its consequences for the state of skeletal muscles increase with age [51].

Naked mole rat is commonly compared with mice as they both are small rodents approximately of the same size and have rather similar constitution. Our studies have shown that mitochondrial apparatus degeneration in skeletal muscles of mice (unpublished date) and rats [52] is observed by the age of 2-2.5 years. Integrated system of mitochondrial reticulum present as a network of branched extended mitochondria in the isotropic band of the muscle fiber is impaired. Only isolated elongated mitochondria of irregular convoluted shape could be observed (Supplementary Figure 2A, 2B). At the same time association of mitochondria in the isotropic band areas into integral mitochondrial system along the whole muscle fiber through longitudinal strands of mitochondria located along the bundles of myofibrils is also lost. There are mostly only small mitochondria observed on the longitudinal sections of the muscle fiber (Supplementary Figure 3A, 3B). Morphometric analysis also shows decreased ratio of the total mitochondrial area to the total area of muscle fiber. At the same time, there is a more than 5-fold increase in the proportion of the mitochondria area compared to the total muscle fiber area in the skeletal muscle (4.8±0.4% to 25.7±3%) of the naked mole rat aged 6 months to 11 years without ultrastructural signs of aging.

It is well known that one of the characteristic signs of aging common to almost all animals is sarcopenia, an age-dependent degradation of structural and functional condition of skeletal muscles associated with both impaired redox processes and decreased muscular work which is largely related to energy metabolism in muscle tissue. The leading role of mitochondrial apparatus in this process is generally acknowledged: its degradation is of pivotal role along with mitochondrial dysfunction [1, 2, 9, 11, 12–22]. Our studies have shown that even at 11 years of age naked mole rats don’t have any pathological changes of skeletal muscle mitochondria, but on contrary substantial growth and development of the mitochondria.

We suppose that specific structure of mitochondrial apparatus developed in the skeletal muscle of naked mole rats by the age of 11 years ensures the appropriate level of oxidation-reduction processes in muscles preventing performance decrease and sarcopenia development.

## MATERIALS AND METHODS

### Animals

### Naked mole rats

Five groups of naked mole rats (1-week, 6-month-, 5-year-, 7-year- and 11-year-old) were used. Each group contained four animals. Naked mole rat colonies are kept at the Leibniz-Institute for Zoo and Wildlife Research (Berlin) in artificial plexiglass labyrinths. The temperature in the system was maintained at 26-29° C, and relative humidity was 60-80%. The boxes contained wooden litter, small twigs, and pieces of paper. Fresh food was available daily without restrictions and included sweet potatoes, carrots, apples, fennel, groats with vitamins and minerals, and oat flakes. Experiments were approved by the Ethics Committee of Landesamt für Gesundheit und Soziales, Berlin, Germany (#ZH 156; G 0221/12; T 0073/15).

### Electron microscopy

This part of the study was done in Belozersky research institute of Physico-chemical Biology, Lomonosov Moscow State University, Moscow. For this examination, tissue of the *m. gracilis* and medial ventrum of *m. quadriceps femoris* wall were excised and fixed with 3% glutaraldehyde solution (Sigma Aldrich, USA) in 0.1 M phosphate buffer (pH 7.4) for 2 h at 4° C. Further it was fixed with 1% osmium tetroxide for 1.5 h and then dehydrated in alcohol series with increasing alcohol concentrations of 50, 60, 70, 80 and 96% (70% alcohol contained 1.4% uranylacetate; Serva, Germany) to enhance contrast. After that, samples were embedded in Epon812 epoxy resin. A series of ultrathin sections was prepared with an ultra microtome (Leica, Austria) and stained with lead. The obtained preparations were imaged and photographed under a JEM1400 electron microscope (JEOL, Japan) operating at the accelerating voltage of 100 kV and beam current of 65 μA equipped with a QUEMESA camera(Olympus, USA) and processed with the software provided with the electron microscope (EMSIS GmbH, Germany).

### Morphometry and statistical analysis

For morphometric examination, ten electron microscopic photographs (magnification ×1500) for each group of animals were selected. In these photos, mitochondria of muscle fibers were marked using the Adobe Photoshop (Adobe®, San Jose, USA) graphical editor, and the number of mitochondrial cross-sections was counted using the Count tool. Several parameters were calculated using the Photoshop analysis package: (1) the number of mitochondrial cross-sections per square micrometer of muscle fibers; (2) the average area of mitochondrial cross-sections; (3) the ratio of total area of mitochondrial cross-sections in one cut to total area of the muscle fiber, which determines the volume fraction of mitochondria in the fiber volume.

## Supplementary Material

Supplementary Figures
