# Supplementary material for: Progressive reorganization of mitochondrial apparatus in aging skeletal muscle of naked mole rats (Heterocephalus glaber) as revealed by electron microscopy: potential role in continual maintenance of muscle activity
Source: Aging (Albany NY). 2021 Nov 28;13(22):24524–41. doi: 10.18632/aging.203720 (PMC8660618; doi:10.18632/aging.203720)
Supplement: Supplementary Figures [file aging-13-203720-s001.pdf]

SUPPLEMENTARY FIGURES

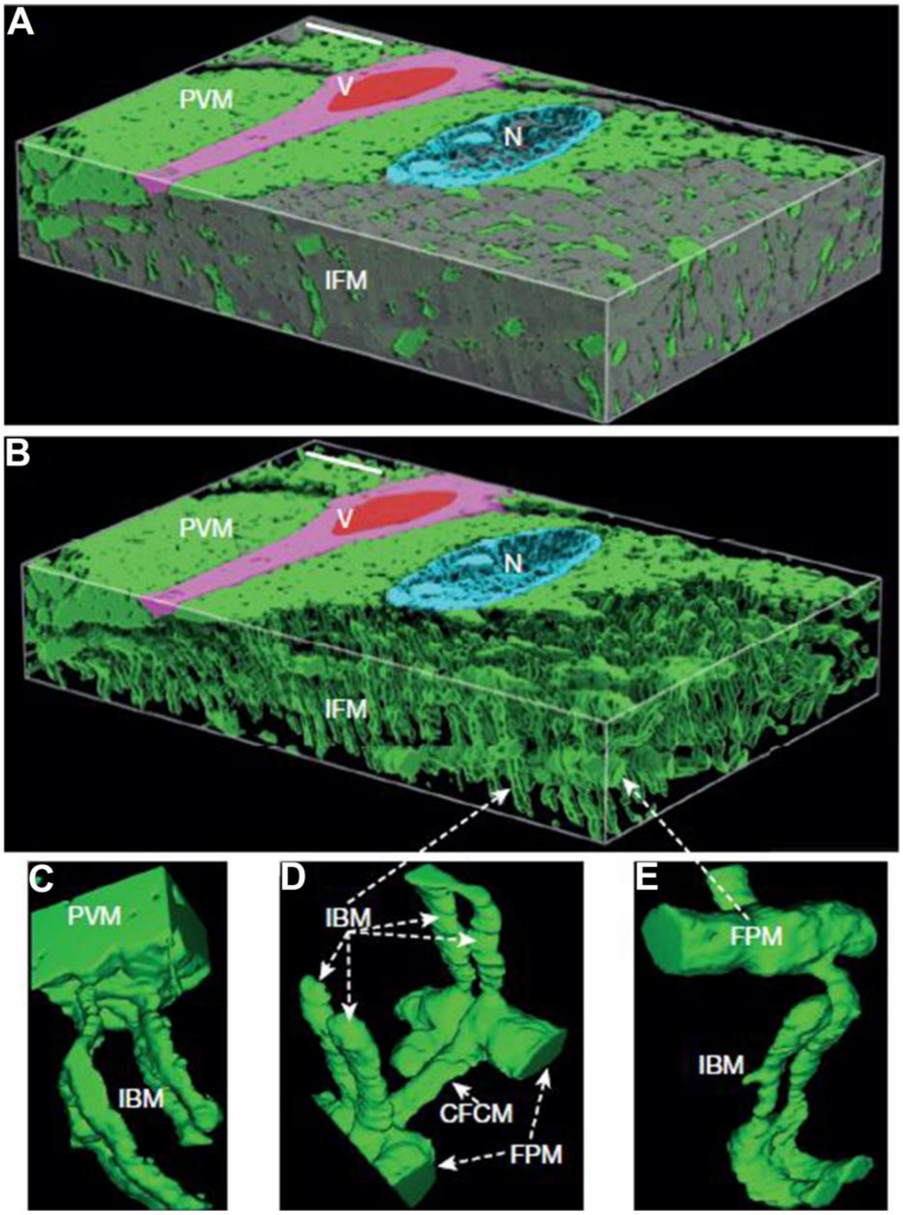

Supplementary Figure 1. Muscle mitochondria form highly connected networks. (**A**) 3D surface rendering of 25.53*24.06*4.23 µm FIB-SEM volume segmented to show spatial relationships between mitochondria (green) and other structures (nucleus (N), cyan; capillary (V), magenta; erythrocyte, red; myofibrils, grey). (**B**) Removing myofibrils highlights different morphologies within intrafibrillar mitochondrial (IFM) network. (**C**–**E**) Zooming in reveals projections from paravascular mitochondria (PVM) into I-band mitochondria (IBM) (**C**), and numerous interactions between IBM and cross-fibre connection mitochondria (CFCM) (**D**) and fibre parallel mitochondria (FPM) (**D**, **E**). Scale bars, 3 µm. Representative of eight separate volumes analysed from four animals. (From Glancy et al. [42]).

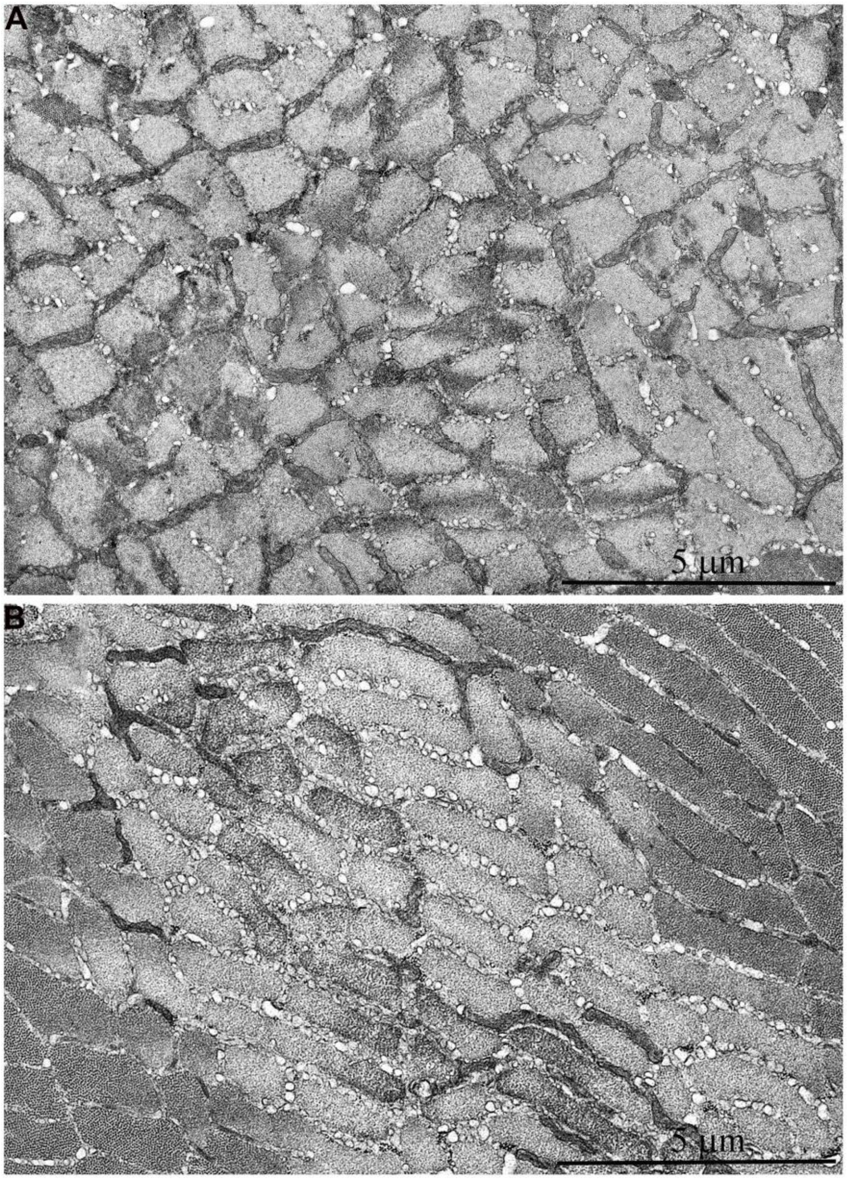

Supplementary Figure 2. Ultrastructure of the skeletal muscle mitochondria of the C57BL/6 mouse on a cross-section. (**A**) At the age of 2.5 months; (**B**) at the age of 2.5 years.

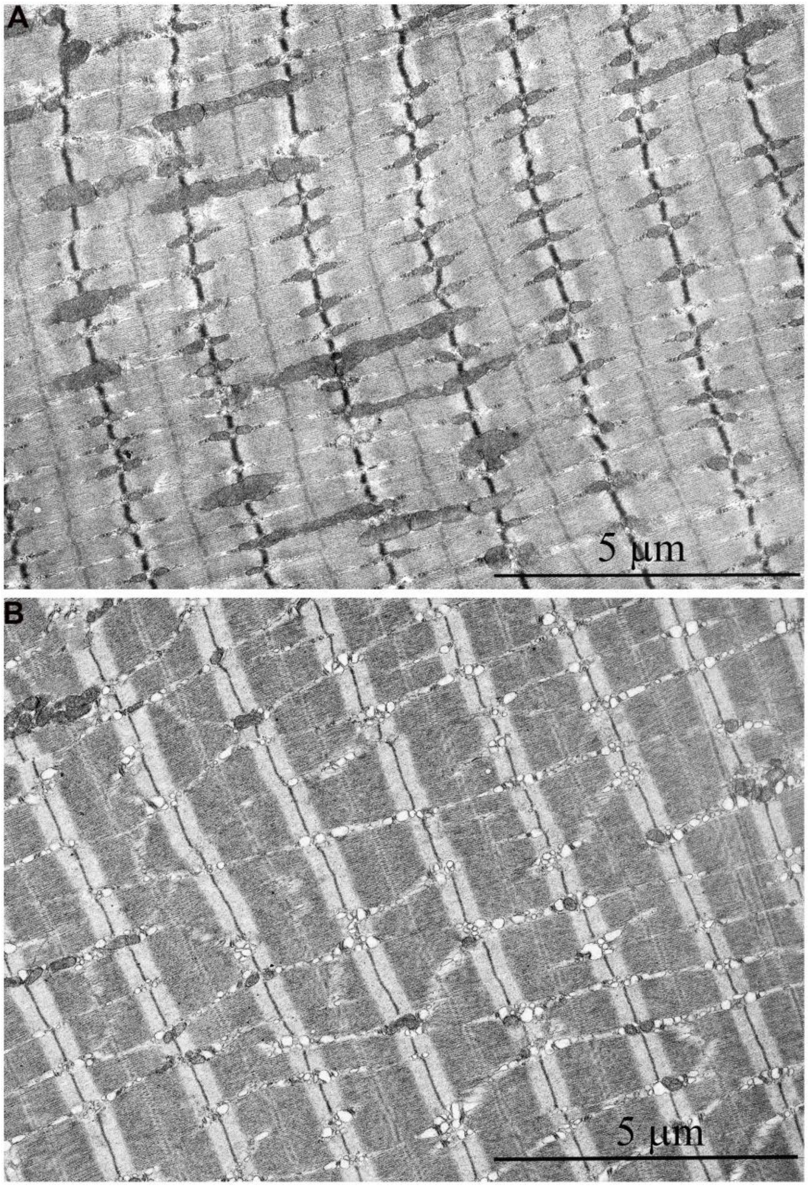

Supplementary Figure 3. Ultrastructure of the skeletal muscle mitochondria of the C57BL/6 mouse on a longitudinal section. (**A**) At the age of 2.5 months; (**B**) at the age of 2.5 years.
